# *Yersinia pestis* Intracellular Parasitism of Macrophages from Hosts Exhibiting High and Low Severity of Plague

**DOI:** 10.1371/journal.pone.0042211

**Published:** 2012-07-27

**Authors:** Duraisamy Ponnusamy, Kenneth D. Clinkenbeard

**Affiliations:** Department of Veterinary Pathobiology, Center for Veterinary Health Sciences, Oklahoma State University, Stillwater, Oklahoma, United States of America; Wayne State University, United States of America

## Abstract

**Background:**

*Yersinia pestis* causes severe disease in natural rodent hosts, but mild to inapparent disease in certain rodent predators such as dogs. *Y. pestis* initiates infection in susceptible hosts by parasitizing and multiplying intracellularly in local macrophages prior to systemic dissemination. Thus, we hypothesize that *Y. pestis* disease severity may depend on the degree to which intracellular *Y. pestis* overcomes the initial host macrophage imposed stress.

**Methodology/Principal Findings:**

To test this hypothesis, the progression of *in vitro* infection by *Y. pestis* KIM62053.1+ of mouse splenic and RAW264.7 tissue culture macrophages and dog peripheral blood-derived and DH82 tissue culture macrophages was studied using microscopy and various parameters of infection. The study showed that during the early stage of infection, intracellular *Y. pestis* assumed filamentous cellular morphology with multiple copies of the genome per bacterium in both mouse and dog macrophages. Later, in mouse macrophages, the infection elicited spacious vacuolar extension of *Yersinia* containing vacuoles (YCV), and the filamentous *Y. pestis* reverted to coccobacillary morphology with genomic equivalents approximately equaling colony forming units. In contrast, *Y. pestis* infected dog macrophages did not show noticeable extension of YCV, and intracellular *Y. pestis* retained the filamentous cellular morphology for the entire experiment in DH82 cells or were killed by blood-derived macrophages. In addition, during the later stage of infection, *Y. pestis* infected mouse macrophages exhibited cell lysis whereas dog macrophages did not.

**Conclusion/Significance:**

Overall, these results support our hypothesis that *Y. pestis* in mouse macrophages can overcome the initial intracellular stress necessary for subsequent systemic infection. However, in dogs, failure of *Y. pestis* to overcome macrophage imposed stress may result in mild or in apparent disease in dogs.

## Introduction

The etiological agent of plague, the Gram-negative bacterium *Yersinia pestis*, causes severe disease in natural rodent hosts such as mice, ground squirrels and prairie dogs, but mild to in apparent disease in some rodent predators such as domestic dogs and coyotes [1]–[4]. *Y. pestis* is maintained in rodent populations in endemic areas as a flea transmitted disease [1], [2]. Rodent predators acquire the infection by either ingestion of infected rodents or via bite of *Y. pestis* infected rodent fleas [5]–[8]. The mechanism underlying the difference in disease severity of rodents and canines to infection by *Y. pestis* is not understood.

The high lethality of *Y. pestis* infection in rodents is demonstrated by the periodic extinction of local rodent populations during seasonal plague epizootics as well as by high mortality rates in experimental infection studies in rodents. In brown Norway rats, intradermal inoculation of 5×10^2^ CFU per animal, to mimic the natural flea bite transmission, caused 100% mortality within 3 to 15 days depending on the site of inoculation [9]. Shortly after the intradermal injection, reddish papular eruptions occur at the site, which is followed by enlargement of local lymph nodes, septicemia and death of infected animals [9]. Similar disease progression and mortality was observed for infection by parenteral inoculation or infected flea bites in mouse models, with infected animals succumbing to the disease within 2 to 8 days post-infection depending on the inoculation dose [10], [11]. Following the bite of an infected flea or experimental injection, subcutaneous *Y. pestis* are phagocytized by tissue neutrophils and macrophages [1], [12], [13]. *Y. pestis* are readily killed by neutrophils, but this initial neutrophilic restriction of *Y. pestis* is only effective for the first few hours post-infection; thereafter, expression of anti-phagocytic factor F1-antigen by *Y. pestis* reduces this process [1], [14], [15]. In contrast to killing by neutrophils, *Y. pestis* survives inside rodent macrophages during the early stage of infection [1]. Phagosomes containing *Y. pestis* mature from early endosomes to phagolysosomes, but the bacteria are able to survive and multiply, thereby allowing dissemination while evading host innate immunity [16]–[22]. While residing in phagolysosomes, *Y. pestis* expresses various stress response and virulence genes such as type-III secretion system, and F1- and pH 6-antigens; and modifies the phagolysosomes into spacious vacuoles to adapt for progression of the infection by systemic dissemination [23]–[26]. Depletion of macrophages in mice by treatment with clodronate-liposomes diminished the severity of infection by *Y. pestis* as indicated by a marked reduction in lesions in spleens and livers of inoculated animals [27]. Overall, infection studies support the role of *Y. pestis* infection of host macrophages in establishing local infection and systemic dissemination of infection following introduction of *Y. pestis* into the host through flea bites [15].

In contrast to flea transmission in natural rodent hosts, rodent predators acquire *Y. pestis* primarily by ingestion of infected rodents [5], [6]. Some rodent predators such as dogs and coyotes are less susceptible for developing severe disease from infection by *Y. pestis*, whereas other rodent predators such as black-footed ferrets and domestic cats are highly susceptible to infection by *Y. pestis*, developing lymphadenopathy of the lymph nodes of the head or neck and subsequent systemic dissemination similar to the disease progression observed in rodents [28], [29]. In experimental infection studies in which cats ingested infected rodents, 80 to 100% of exposed cats developed clinical illness with 38 to 42% mortality [28], [30]. Although domestic cats and dogs in endemic regions likely have similar rates of exposure to *Y. pestis* infected rodents, there are many more case reports in the literature of clinical disease in cats than in dogs, suggesting that the latter experience less severe disease [28], [30]–[32]. In one of the few case reports of plague in dogs, the clinical signs of mild fever, malaise, stomatitis, and transient submandibular lymph node swelling were observed [5]. Unlike experimentally infected cats, dogs infected experimentally with *Y. pestis* through either oral or subcutaneous route exhibited only mild clinical signs of short duration with no mortality [33]. The mechanism by which some species exhibit less severe disease from *Y. pestis* infection than others may be related to how well *Y. pestis* overcomes stress associated with intracellular parasitism of host macrophages. Although natural rodent hosts and canid rodent predators are infected via different routes, it appears likely that *Y. pestis* utilizes the same mechanism of evasion of the host innate immunity by intracellular parasitism of host macrophages and subsequent systemic dissemination in hosts which exhibit both high and low severity of disease.

During *Y. pestis* infection, macrophages can employ a wide spectrum of antimicrobial defenses including phagolysosomal acidification; enzymatic actions from cathepsins, lipase, nuclease and glycosidase; reactions of oxidizing agents and reactive oxygen species; and effects of cationic peptides, nitric oxide and reactive intermediates of nitric oxide. Furthermore, by the action of natural resistance-associated macrophage protein (NRAMP), many phagolysosomal metal ions such as iron, calcium, magnesium and manganese are sequestered, making the organelle very hostile for survival and growth of intracellular *Y. pestis* [34], [35]. In order to adapt to and survive in this harsh intracellular environment, *Y. pestis* relies on various general stress regulators and other mechanisms to reduce the phagolysosomal antimicrobial activities [17], [18], [36], [37].

Based on these observations, we hypothesized that differences in severity of disease from *Y. pestis* infection between high severity in rodents and less severe disease in dogs may be related to whether *Y. pestis* is able to overcome macrophage imposed stress during the intracellular parasitism phase of infection. In agreement with our hypothesis, *Y. pestis* in mouse macrophages exhibited morphological plasticity, survived during the entire experimental period of 27.5 h, mediated alternation in phagolysosomes, and caused cell lysis of the infected macrophages. However, in dog macrophages, intracellular *Y. pestis* either remained in a stressed state or were killed by infected macrophages without inflicting damage to these macrophages.

## Results

### Intracellular parasitism of mouse and dog macrophages by *Y. pestis*

To examine the hypothesis that disease severity in different host species may be related to how well *Y. pestis* overcomes macrophage imposed stress, we examined the morphological changes of *Y. pestis* in primary macrophages derived from mouse spleen and dog peripheral blood. These macrophages were infected with *Y. pestis* strain KIM62053.1+, and bacterial and macrophage morphology observed by light microscopy and transmission electron microscopy (TEM) over a 27.5 h infection period for which the 0 time was the initiation of infection. *In vitro Y. pestis* infections of mouse macrophages have been previously reported [38], but infections of dog macrophages have not.

**Figure 1:**
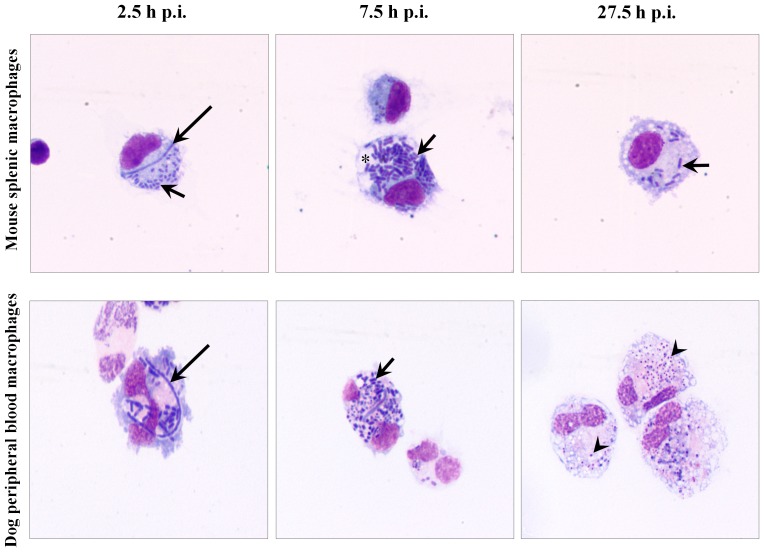
Intracellular *Y. pestis* in mouse and dog primary macrophages. *Y. pestis* strain KIM6+ infected primary macrophages from mouse spleen (top row) and dog peripheral blood (bottom row) were sampled at 2.5, 7.5 and 27.5 h p.i. and observed under light microscope after staining with Wright Giemsa stain. Long arrows, short arrows and arrow heads represent filamentous, coccobacillary and degraded coccobacillary *Y. pestis*, respectively. Asterisk indicates spacious vacuolar extension of phagolysosomes. Images are at a 1,000× magnification.

**Figure 2:**
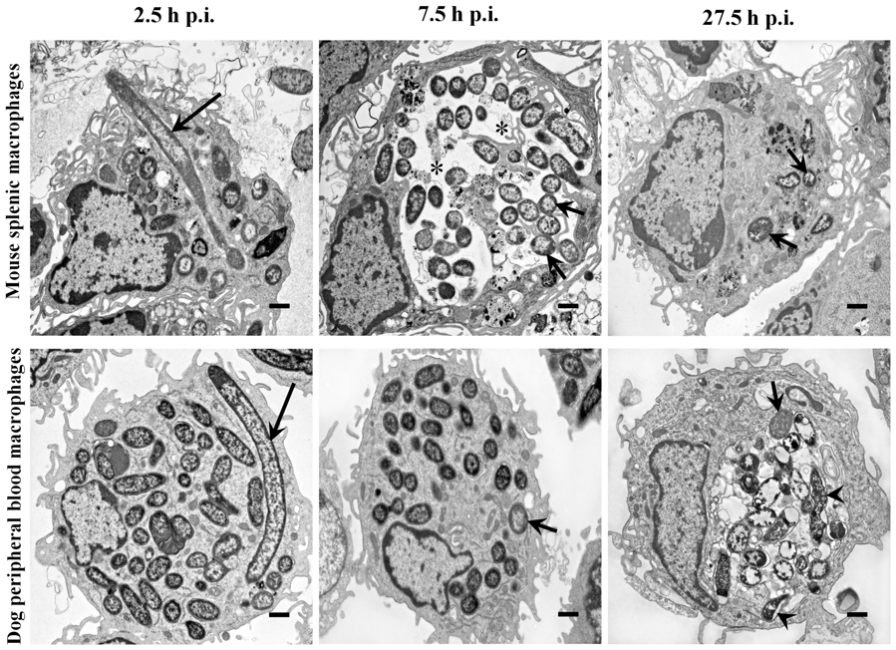
Ultrastructural features of intracellular *Y. pestis* and infected mouse and dog primary macrophages. Primary macrophages from mouse spleen (top row) and dog peripheral blood (bottom row) infected with *Y. pestis* strain KIM6+ were glutaraldehyde fixed at 2.5, 7.5 and 27.5 h p.i. and examined using TEM. Long arrows, short arrows and arrow heads represent filamentous, coccobacillary and degraded coccobacillary *Y. pestis*, respectively. Asterisks indicate spacious vacuolar extension of phagolysosomes. The images are at a 6,000× magnification, and the cross bars represent 1 µm.

Mouse splenic macrophages were readily infected with *Y. pestis* with macrophages containing 18.0±13.2 *Y. pestis* per infected macrophage at 2.5 h p.i. Intracellular *Y. pestis* were primarily coccobacilli housed in tight YCV (Figs. 1 and 2). However, 6% of the intracellular *Y. pestis* exhibited filamentous bacterial morphology with a mean filament length of 6.8 µm and partial or no visible septation. At 7.5 h p.i., 82% of the mouse macrophages exhibited spacious YCVs and the number of *Y. pestis* per macrophage increased by 115%, but filamentous forms were not observed. At 27.5 h p.i., few splenic macrophages were observed, suggesting that macrophages lost viability between 7.5 and 27.5 h p.i. The splenic macrophages present at 27.5 h p.i. appeared larger with foamy cytoplasm, and intracellular *Y. pestis* had a bipolar rod appearance. Overall, microscopic detection of filamentous *Y. pestis* in mouse macrophages at the initial stage of infection may represent an adaptation of the bacterium to macrophage imposed stress. Subsequently by extending YCVs into spacious compartments at 7.5 h p.i., intracellular *Y. pestis* may be released from the macrophage imposed stress, return to normal coccobacillary bacterial morphology, and multiplied intracellularly. *Y. pestis* infected mouse macrophages lost viability by 27.5 h p.i., likely releasing viable and stress-adapted *Y. pestis*.

Dog peripheral blood derived macrophages were also readily infected with *Y. pestis* with macrophages containing 23.9±13.9 *Y. pestis* per infected macrophage at 2.5 h p.i. Similar to mouse splenic macrophage infections, 5% of the intracellular *Y. pestis* in dog peripheral blood derived macrophages exhibited filamentous bacterial morphology with a mean filament length of 8.4 µm, and *Y. pestis* were contained within tight YCVs (Figs. 1 and 2). At 7.5 h p.i., the number of intact coccobacilli per macrophage increased by 38% from 2.5 h p.i., compatible with transition of filamentous *Y. pestis* to coccobacilli by cellular division, but unlike mouse macrophages the bacteria continued to be sequestered in tight YCVs. At 27.5 h p.i., intracellular *Y. pestis* appeared to be housed in YCV with double membranes in 80% of the infected macrophages; however, most of these intracellular *Y. pestis* were coccobacilli which exhibited irregular morphology consisting of vacuolation and uneven distribution of electron dense aggregates on TEM cross sections, compatible with killing of the bacteria by the dog macrophages. In contrast to the spatial extension of YCV in *Y. pestis* infected mouse macrophages, changes in dog macrophage morphology were restricted to increased foamy cytoplasm at 7.5 and 27.5 h p.i. These findings suggest that in dog macrophages, although intracellular *Y. pestis* exhibit similar stress morphology as in mouse splenic macrophages, *Y. pestis* failed to extend YCV at 7.5 h p.i., possibly facilitating subsequent killing of *Y. pestis* by these macrophages.

### Intracellular *Y. pestis* parasitism of mouse and dog macrophage cell lines

**Figure 3:**
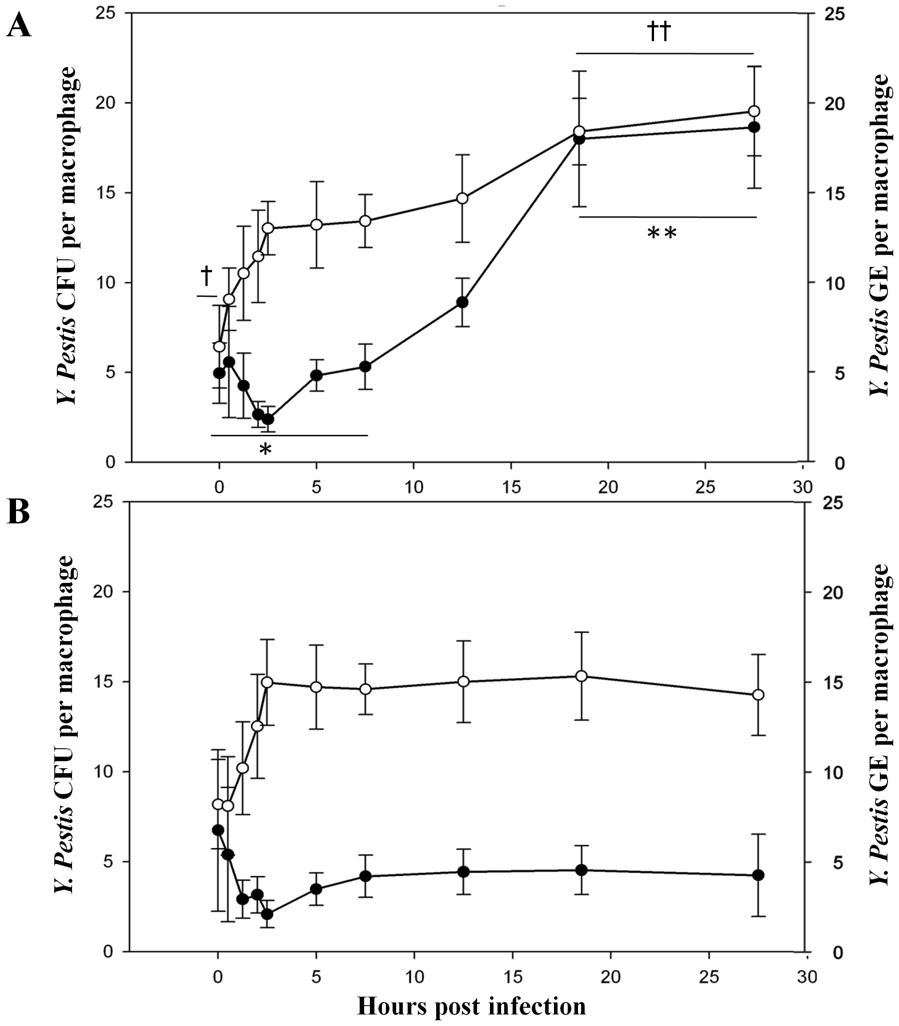
Intracellular CFUs and GEs per tissue culture macrophage at various times p.i. CFUs (•) for culturable and GEs (○) for total *Y. pestis* per RAW264.7 (A) and DH82 (B) were calculated for various intervals from 0 to 27.5 h p.i. using microplate based colony counting and qPCR methods described in Materials and Methods section. The results are expressed as mean ± SEM (n = 3). Means and variances of CFUs or GEs per macrophage at different p.i. intervals for RAW264.7 and DH82 cells were analyzed initially using one-way ANOVA. No significant differences were found between means within groups denoted * for CFUs per macrophage for 0 to 7.5 h p.i., † GEs per macrophage for 0 to 0.5 h p.i., ** for CFUs per macrophage for 18.5 to 27.5 h p.i., or †† GEs per macrophage for18.5 to 27.5 h p.i.. The groups were then tested for significant differences at p = 0.05 between groups using a ‘Tukey HSD’ post hoc method. The means for * CFUs per macrophage for 0 to 7.5 h p.i. differed from the means for ** CFUs per macrophage for 18.5 to 27.5 h p.i. at the p<0.05 level, and the means for † GEs per macrophage for 0 to 0.5 h p.i. differed from the means for the †† GEs per macrophages for 18.5 to 27.5 h p.i. at the p<0.05 level.

**Figure 4:**
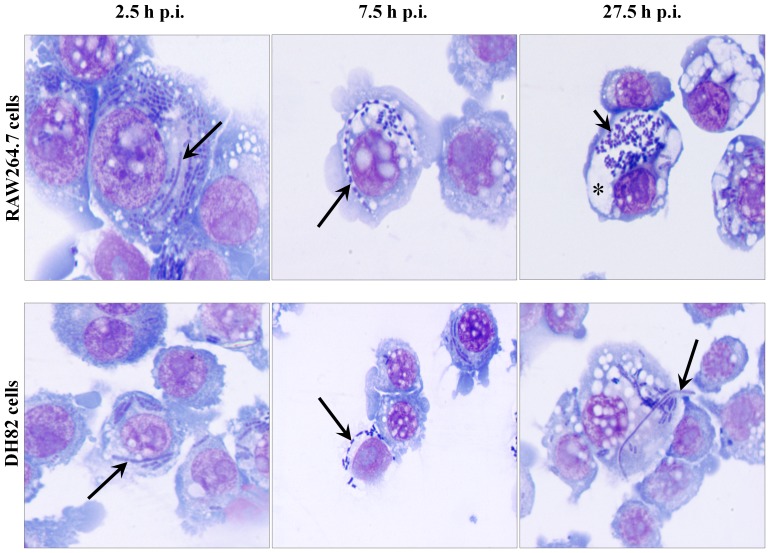
Morphological features of intracellular *Y. pestis* and of infected mouse and dog tissue culture cells. *Y. pestis* strain KIM6+ infected RAW264.7 (top row) and DH82 (bottom row) cells were sampled from 2.5 to 27.5 h of p.i. and observed under light microscope by staining with Wright Giemsa stain. Filamentous and coccobacillary forms of intracellular *Y. pestis* and spacious phagolysosomal extensions were determined at a 1,000× magnification. Long and short arrows indicate filamentous and coccobacillary form of intracellular *Y. pestis*, respectively. Asterisk indicates spacious vacuolar extension of phagolysosome.

**Figure 5:**
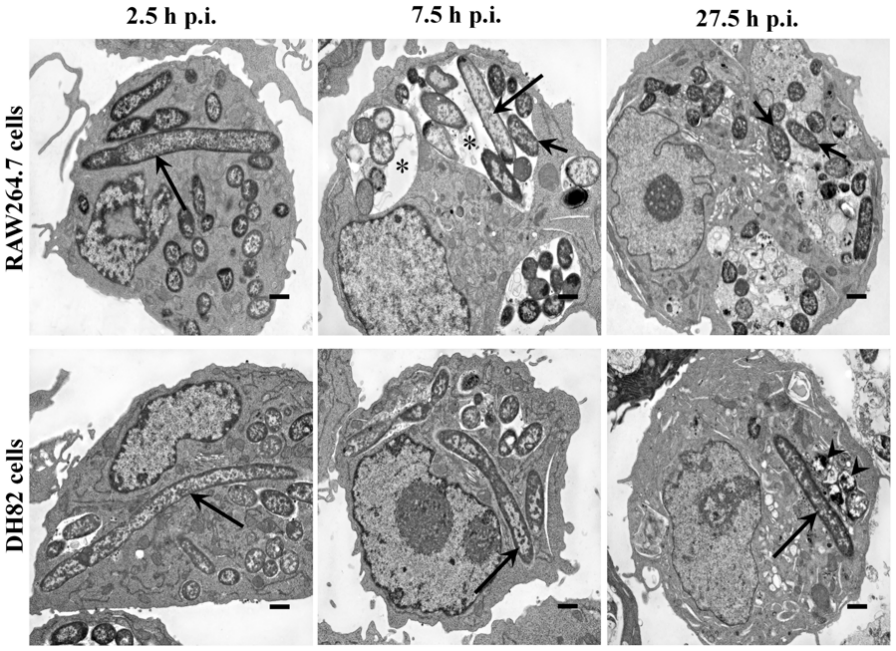
Ultrastructural features of intracellular *Y. pestis* and infected mouse and dog tissue culture cells. *Y. pestis* strain KIM6+ infected RAW264.7 (top row) and DH82 (bottom row) cells were sampled at 2.5, 7.5 and 27.5 h p.i. and examined in transmission electron microscope. Long and short arrows indicate filamentous and coccobacillary form of intracellular *Y. pestis*, respectively. Arrow heads indicate degraded intracellular *Y. pestis*. Asterisks indicate spacious vacuolar extension of phagolysosomes. The images are at a 6,000× magnification, and cross bars represent 1 µm.

In order to better quantify and compare intracellular parasitism of *Y. pestis* in macrophages from hosts exhibiting different severity of disease, colony forming units (CFUs) and genomic equivalents (GEs) per macrophage were measured and light and electron microscopic images obtained for *in vitro Y. pestis* infection of mouse RAW264.7 and dog DH82 tissue culture macrophages. Like the primary mouse and dog macrophages, the tissue culture macrophages experienced high infection rates of >90% with 13.8±9.1 and 10.1±5.8 *Y. pestis* bacteria per RAW264.7 and DH82 macrophage, respectively, at 2.5 h p.i. (Table 1). However, during this initial infection interval from 0 to 2.5 h, both RAW264.7 and DH82 cells exhibited a trend to lower *Y. pestis* CFUs but higher GEs per macrophage (Fig. 3A and 3B), with the GE more closely reflecting the level of intracellular bacteria observed by microscopy in macrophages. Both Wright Giemsa stained light microscopic and TEM images at 2.5 h p.i., showed 7–8% of *Y. pestis* exhibited filamentous bacterial morphology (Figs. 4 and 5, Table 1), suggesting that the lower CFUs and accompanying higher GEs may in part reflect the filamentous growth of *Y. pestis* in both macrophage cell lines. This filamentous change was also evidenced under UV-fluorescent microscope in *Y. pestis* strain KIM6+ GFPuv infection of RAW264.7 and DH82 cells at 3 to 4 h p.i. (images not shown). The trend to lower CFUs but higher GEs may also reflect lower efficiency of microbial culture of stressed intracellular *Y. pestis*.

**Table 1:** Morphometric analysis of intracellular *Y. pestis* and tissue culture macrophages from TEM images.

Parameter	RAW264.7 cells			DH82 cells		
**Hours post infection**	**2.5**	**7.5**	**27.5**	**2.5**	**7.5**	**27.5**
**Intact ** ***Y. pestis*** ** per macrophage**	13.8±9.1	16.5±9.7	14.3±7.8	10.1±5.8	7.7±6.7	7.2±6.1
**Filamentous ** ***Y. pestis*** ** per macrophage**	1.0±0.4§§	0.8±0.8†	0.3±0.5§§ ^, ^ †	0.8±0.4	1.2±0.4	1.1±1.4
**% Macrophages with spacious YCV**	13	64	73	18	23	18

† p<0.05;

§§ p<0.01 as analyzed through a one-way ANOVA and Tukey HSD post hoc method.

From 2.5 to 7.5 h p.i., there was a 19% increase in *Y. pestis* per RAW264.7 cells but a decline of 24% of *Y. pestis* per DH82 cells observed (Table 1). This correlated with a trend to higher CFUs with no change in GEs per macrophage for RAW264.7, but no change for either CFUs or GEs for DH82 cells (Fig. 3). The percent of filamentous *Y. pestis* remained about 5% in RAW264.7 cells but increased to 16% in DH82 cells (Table 1). The most remarkable change during this infection period was the increase of spacious YCV in infected RAW264.7 cells with 64% of RAW264.7 cells exhibiting spacious YCV at 7.5 h p.i. (Fig. 5, Table 1).

For RAW264.7 cells between 7.5 to 27.5 h p.i., the spacious YCV were maintained, the observed *Y. pestis* per RAW264.7 cell declined by 9% and filamentous *Y. pestis* declined from 5% to 2% (Table 1). However, the CFUs per macrophage increased significantly and became approximately equal to the GEs per macrophage, suggesting that *Y. pestis* which had been filamentous and/or not culturable at 7.5 h p.i. had undergone cell division to coccobacilli and become culturable at 27.5 h p.i. (Fig. 3, 4 and 5, Table 1). During this 7.5 to 27.5 h p.i. period in DH82 cells, CFUs, GEs and % filamentous *Y. pestis* remained unchanged, but at 27.5 h p.i., 29% of intracellular *Y. pestis* showed loss of viable bacterial morphology in TEM images (Fig. 5). These results suggest that *Y. pestis* in RAW264.7 cells experienced macrophage imposed stress as reflected by the filamentous bacterial morphology and non-culturability, but then overcame this stress coincident with vacuolar extension of YCV and return to coccobacillary morphology and culturability. But in DH82 cells, *Y. pestis* likely failed to overcome the macrophage imposed stress resulting in loss of viability as evidenced in TEM images at 27.5 h p.i.

### Intracellular *Y. pestis* morphology in antibiotic-free media

**Figure 6:**
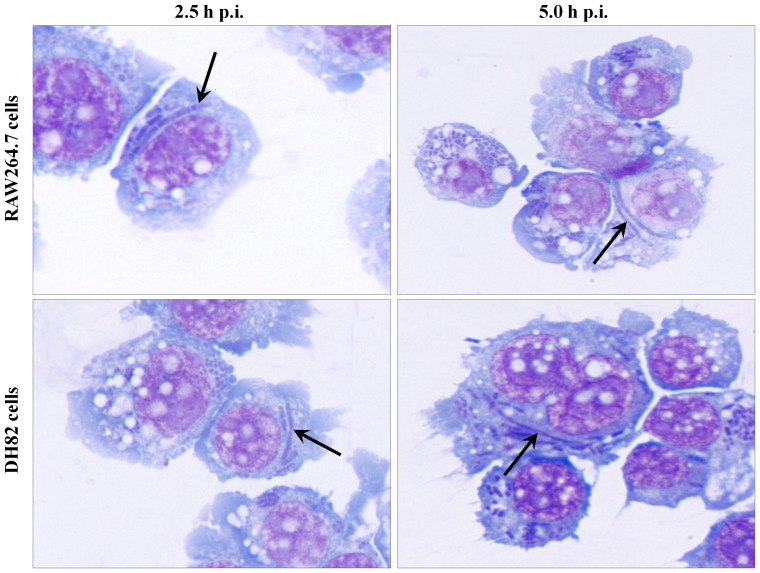
Morphological features of intracellular *Y. pestis* in antibiotic-free media. RAW264.7 and DH82 cells infected with *Y. pestis* strain KIM6+ for 30 min in RPMI-1640 with 10% FBS media, washed with PBS to remove extracellular bacteria, and then incubated in HBSS with 10% FBS were sampled at 2.5 and 5 h p.i. for light microscopic examination by staining with Wright Giemsa stain. Arrows indicate filamentous *Y. pestis*. Images are presented at 1,000× magnification.

Exposure of bacteria to gentamicin has the potential to induce aberrant bacterial morphology including filamentous forms [39]. To assess whether intracellular *Y. pestis* in macrophages exhibited filamentous morphology independent of gentamicin used for killing extracellular bacteria in the infection protocol, RAW264.7 and DH82 cells infected with *Y. pestis* were washed to remove extracellular *Y. pestis* rather than being treated with gentamicin. The washed infected RAW264.7 and DH82 cells were then incubated in Hank's Basic Salt Solution (HBSS) and 10% fetal bovine serum (FBS) to reduce growth of any residual extracellular *Y. pestis*, and the intracellular *Y. pestis* were observed by light microscopy for filamentous morphology. In the absence of gentamicin treatment, filamentous *Y. pestis* were observed at 2.5 and 5.0 h of infection in RAW264.7 and DH82 cells (Fig. 6), indicating that gentamicin was likely not responsible for the observed filamentous morphology of *Y. pestis* in macrophages.

### Cytotoxicity of *Y. pestis* infected RAW264.7 and DH82 cells

**Figure 7:**
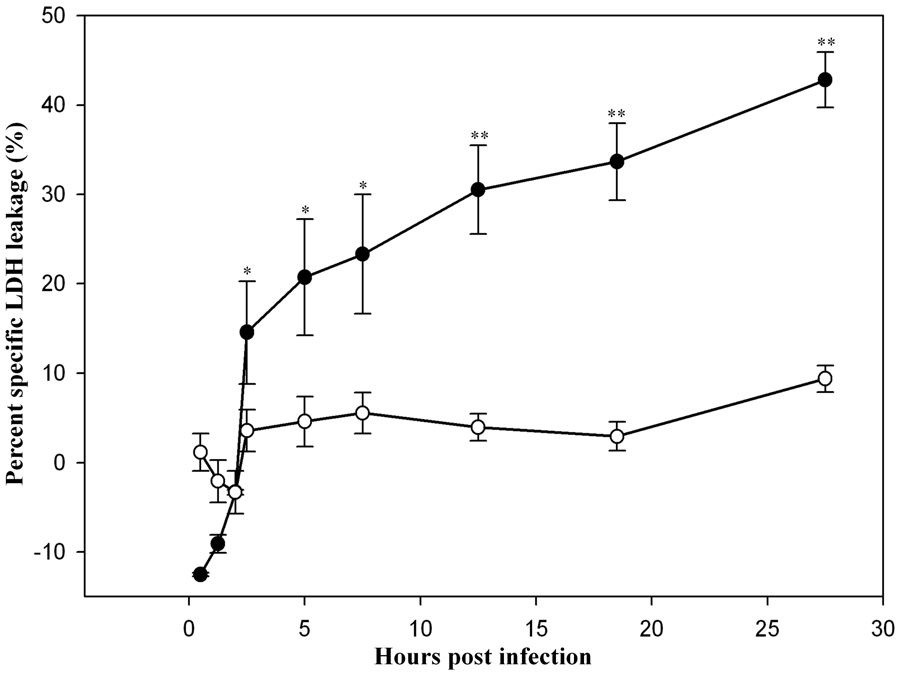
Cytotoxicity assay of mouse and dog tissue culture cells infected with *Y. pestis*. The specific leakage of the macrophage cytoplasmic enzyme LDH associated with *Y. pestis* infection of RAW264.7 (•) and DH82 (○) cells was determined. The data are shown as means ± SEM (n = 3). Asterisks on error bars represent, at the respective interval, statistical difference (_*_, p<0.05; _**_, p<0.01) between the means percentage specific LDH leakage of RAW264.7 and DH82 cells in Student's t-test.

As previously reported for mouse J774A.1 macrophage cell line, *Y. pestis* infection is associated with loss of macrophage viability [40]. Using the trypan blue dye exclusion method, RAW264.7 cells experienced an approximate 50% loss of cell viability, but only a 20% loss of cell viability was observed for *Y. pestis* infection of DH82 cells. To further characterize this loss of viability, lactate dehydrogenase leakage from infected macrophages was also determined. As shown in Figure 7, RAW264.7 cells infected with *Y. pestis* experience rapid cell lysis as indicated by specific LDH leakage during the initial 2.5 h p.i. period, and then slower, steady lysis through 27.5 h p.i. resulting in 45% lysis of infected cells as compared with uninfected control RAW264.7 cells exhibiting 0% cell lysis. In contrast, *Y. pestis* infection of DH82 cells exhibited cell lysis of approximately 10% for the entire infection period, which was not statistically significant from that for uninfected DH82 cells. These results show that *Y. pestis* infection of RAW264.7 cells causes cell lysis, which may release *Y. pestis* to the extracellular space in infected animals potentially initiating septicemic plague.

### Intracellular*Yersina enterocolitica* infection of mouse and dog macrophage cell lines

We observed that *Y. pestis* overcame macrophage imposed intracellular stress in mouse but not dog macrophages. To assess whether this response was specific for *Y. pestis* compared to another, less pathogenic yersiniae, RAW264.7 and DH82 cells were infected with *Yersinia enterocolitca*. *Y. pestis* and *Y. enterocolitca* share similar genomic content and are both facultative intracellular pathogens [20], [41]. As shown in Table 2, *Y. enterocolitica* infected both RAW264.7 and DH82 cells at similar levels of CFUs/macrophage, and this level of infection declined in both mouse and dog macrophages during the incubation period demonstrating no significant differences in intracellular parasitism of mouse versus dog macrophages in contrast to the difference in host macrophage infectivity observed for *Y. pestis*.

**Table 2:** Comparison of intracellular growth of *Y. enterocolotica* in mouse and dog origin tissue culture macrophages.

Incubation Time	Bacteria per Macrophage‡	
	RAW 264.7 Cells	DH82 Cells
2.5 h	4.7±1.1	4.2±1.0
7.5 h	3.1±0.7	3.5±0.8
27.5 h	0.5±0.1	0.6±0.2

‡ n = 3.

## Discussion

Although *Y. pestis* infects a wide range of animal species, disease severity differs from species to species. Rodents are natural hosts and suffer severe disease with high mortality, but some rodent predators such as dogs and coyotes experience only mild or in apparent disease [1]–[4], [33], [42], [43]. This variation in disease severity among susceptible hosts may be related to the extent to which *Y. pestis* overcomes the initial host macrophage mediated stress. The host macrophages are the primary cells used by *Y. pestis* to evade host innate immune mechanisms and to multiply and disseminate systemically during the early stage of infection [1], [13], [15]–[21], [27]. The goal of our experimental study was to better understand *Y. pestis* responses to infection of macrophages from mice and dogs, as hosts experiencing severe or mild disease, respectively. We hypothesized that *Y. pestis* in macrophages of severely affected hosts likely overcome the intracellular antimicrobial stresses and multiplies intracellularly, whereas, in macrophages of hosts exhibiting only mild disease, *Y. pestis* fails to overcome the intracellular defense mechanisms and are eventually killed by these macrophages.

In our study, we found that a fraction of intracellular *Y. pestis* in host macrophages assumes filamentous bacterial morphology with multiple genome copies per bacterium likely induced as a result of macrophage associated stress. This filamentous morphologic change was frequently noted during the early stage of infection in both primary and tissue culture macrophages of both mice and dogs (Figs. 1, 2, 4 and 5, Table 1). Similar filamentous stress response have been observed for uropathogenic *E. coli* in mouse urinary bladder epithelial cells, *Legionella* spp. in Vero cells, *Mycobacterium tuberculosis* in human macrophage cell line THP-1, and *Salmonella enterica* serovar Typhimurium in mouse bone marrow and RAW264.7 macrophages [44]–[49]. The antibiotic gentamicin used in our infection protocol is also known to induce filamentous changes in exposed bacteria [29]. However, in our study, when gentamicin was omitted from the infection protocol, filamentous *Y. pestis* were still observed supporting intracellular stress as the cause rather than gentamicin.

*Y. pestis* filamentous morphologic response in macrophages may be an adaptive strategy to cope with intracellular antibacterial defense mechanisms in addition to stress relievers such as general stress regulation and inhibition of acidification of YCV [17], [36], [38]. For *Salmonella typhimurium*, filamentous morphologic changes in primary mouse bone marrow macrophages or in RAW264.7 cells are associated with exposure to NADPH oxidase, reactive oxygen species, nitric oxide or proteases associated with phagosome, and furthermore, this morphologic change is positively influenced by IFN-gamma priming of macrophages or presence of cationic antimicrobial peptides during the infection [47], [48], [50]. In support of filamentous morphologic change as a specific adaptive response induced by intracellular stress, the *Y. pestis* SOS DNA repair response to DNA damage occurring intracellularly is also known to cause the bacterium to undergo filamentous morphologic change by interfering with cell division process as observed in other bacteria [46], [51], [52]. Presumably, the filamentous morphologic change observed for intracellular *Y. pestis* is an adaptive strategy to aid survive in the hostile intracellular environment; this structural alteration may prevent the passing of damaged genomic copies to the daughter cells, by extending the time frame for DNA repair [53], [54].

In addition to assumption of a filamentous bacterial morphology, *Y. pestis* also cause spacious vacuolar extension of YCV in mouse splenic and tissue culture macrophages (Figs. 1, 2, 4 and 5, Table 1). This vacuolar extension is consistent with previous report in J774A.1 macrophages infected with *Y. pestis* KIM6 in which YCVs were actively extended by the intracellular *Y. pestis*, and this extension process was dependent on presence of intact copies of PhoP-PhoQ transcriptional regulator [17]. Although the precise molecular mechanism governing this process is unknown, *Y. pestis* mediated extension of YCV likely benefits the bacterium by dilution of phagolysosomal content thereby reducing their antimicrobial activity and associated macrophage imposed stress.

Near coincidently with *Y. pestis* initiated spacious vacuolar extension of YCV in mouse macrophages, the filamentous *Y. pestis* reverted to coccobacillary bacterial morphology (Figs.1, 2, 4 and 5, Table 1). For mouse splenic macrophages, by 7.5 h p.i., the majority of intracellular *Y. pestis* converted back to coccobacilli, which were loosely confined within the spacious YCV. Reversion of *Y. pestis* from filamentous to coccobacillary morphology in mouse macrophages may reflect release of bacteria from intracellular stress. The molecular machinery which mediates the transition from filamentous to coccobacillary morphology is largely unknown; nevertheless, products of *Y. pestis* genes y2313, y2315 and y2316 may play a role in this process. These genes are expressed in mouse macrophage cell line J774A.1 at 4 h p.i, and mutational inactivation was associated with retention of filamentous intracellular *Y. pestis* for at least 24 h of infection [55].

In contrast to YCV changes in mouse macrophages, *Y. pestis* in dog macrophages did not induce vacuolar extension of YCVs, and *Y. pestis* retained its filamentous morphology throughout 27.5 h of infection in dog DH82 macrophages (Fig. 3, 4, and 5, Table 1). This suggests that *Y. pestis* in DH82 cells may not efficiently overcome intracellular stress, and consequently, the bacterium is susceptible to macrophage killing as evidenced by presence of disintegrating coccobacilli in dog YCV at 27.5 h p.i. (Fig. 5). In dog peripheral blood macrophages, *Y. pestis* did convert from filamentous forms to coccobacilli at 7.5 h p.i. but failed to modify YCV and remained in tight YCV, suggesting that intracellular *Y. pestis* are still controlled by macrophage defense mechanisms (Figs. 1 and 2). These coccobacilli eventually died within YCVs due to macrophage killing. This failure of *Y. pestis* to overcome macrophage imposed stress during the initial intracellular parasitism phase of plague may result in less severe disease in dogs and coyotes [1], [33], [42], [43].

Apart from the vacuolar extension of YCV, mouse and dog tissue culture macrophages markedly differed in the loss of macrophage viability from *Y. pestis* infection for the period of 27.5 h p.i. The infected RAW264.7 cells exhibited 45% cell lysis in contrast to approximately 10% for DH82 cells (Fig. 6). The high percentage of LDH leakage in RAW264.7 cells may be associated with spacious extension of YCV leading to lysis of infected macrophages. Furthermore, this finding in RAW264.7 cells agrees with the prevailing speculation that cell lysis is the primary mean of *Y. pestis* release from the infected macrophages [20]. In contrast, restriction of intracellular *Y. pestis* to the stress related filamentous morphology in tight, unmodified YCV for the entire infection may account for the failure of *Y. pestis* to induce lysis of DH82 cells. Similar observations have been made for filamentous *Burkholderia pseudomallei* in THP-1 cells [56].

In conclusion, our results demonstrate that *Y. pestis* in host macrophages assume filamentous morphology during initial stage of exposure to intracellular stress likely as a morphologic adaptation used by the bacterium to prolong its survivability under harsh conditions [54]. These filamentous *Y. pestis* in mouse macrophages eventually returned to coccobacillary morphology, which was associated with active vacuolar extension of YCV and subsequent macrophage cell lysis. In contrast, in dog macrophages, *Y. pestis* was either restricted to the stress-induced filamentous morphology for an extended period of time and then were killed by the macrophages or reverted to coccobacillary morphology but were eventually killed by the macrophages likely due to failure of the bacterium to induce spacious extension of YCV. Therefore, greater severity of infection and fatal outcome of *Y. pestis* infections in rodents and other highly susceptible hosts may be the consequence of *Y. pestis* overcoming macrophage associated stress during the initial phase of infection, whereas less severe disease in some canid rodent predators may be associated with macrophage restriction of the *Y. pestis* stress responsiveness during the intracellular parasitism phase of *Y. pestis* infection.

## Materials and Methods

### Bacterial strains and culture conditions

*Y. pestis* strain KIM62053.1+ *hms* ^+^ *psn* ^+^ *psa* ^−^ (Δ*psa*2053.1) *ybt* ^+^ *lcr* ^−^ derived from KIM62053.1+ (KIM6+) was kindly provided by Dr. Robert Perry, University of Kentucky and used in all experiments except where noted [57]. For fluorescent imaging experiments, KIM62053.1+ was transformed with a modified green fluorescent protein expression plasmid (pGFPuv, Clontech, Mountain View, CA) by electroporation as described elsewhere [58], [59]. *Y. enterocolotica* strain OADDL 91 was obtained from the Oklahoma State University College of Veterinary Medicine infectious diseases laboratory collection. For both Yersinia species, isolated colonies on Brain Heart Infusion (BHI) (Difco, Becton Dickinson Company, Franklin Lakes, NJ) agar plates grown for 36 h at 26°C were inoculated into BHI broth (Difco) and cultured overnight at 26°C with 160 rpm shaking.

### Isolation of mouse splenic macrophages

Splenic macrophages were used instead of blood macrophages due to the difficulty of collecting sufficiently large volumes of blood from mice to isolate the required numbers of macrophages for experimentation. Splenic macrophages were isolated from 7 to 11 week old, female C57BL/6J mice (The Jackson Laboratory, Bar Harbor, Maine). Briefly, spleens from mice euthanized with 70% CO_2_ under an Oklahoma State University Institutional Animal Care and Use Committee approved protocol were collected under sterile conditions in 10–15 mL of Dulbecco's Modified Eagle's Medium (DMEM) (Invitrogen, Grand Island, NY) supplemented with 20% fetal bovine serum (FBS) (Hyclone laboratories, Logan, UT) and 50 µg/mL of gentamicin sulfate (Sigma-Aldrich, St Louis, MO), and therein the same media, spleens were thoroughly minced using a sterile scalpel blade. The resulting cell suspension was passed successively through sterile nylon mesh of 160, 75, 15 µm sizes to yield tissue debris-free homogenous cell suspensions, and thereafter, these cell suspensions were adjusted to 2×10^6^ viable cells/mL in the same media. Finally, 6 mL aliquots of the cell suspensions were cultured overnight in 25 cm^2^, poly-D-lysine coated tissue culture flasks (Becton Dickinson Labware, Franklin Lakes, NJ) at 37°C with 5% CO_2_, followed by removal of non-adherent cells by washing with sterile phosphate buffered saline (PBS). The attached cells were further cultured for 3 days in fresh DMEM, FBS, and gentamicin media as above. Subsequently, the cell layers were changed into RPMI-1640 media (Sigma-Aldrich) supplemented with 20% FBS, 2 g/L sodium bicarbonate and no antibiotic for 2 days prior to use.

### Isolation of dog peripheral blood derived macrophages

Blood was collected from healthy adult dogs via venipuncture under an Oklahoma State University Institutional Animal Care and Use Committee approved protocol and anticoagulated with 10% sodium citrate (Sigma-Aldrich). Anticoagulant peripheral blood was diluted 2-fold with PBS containing 50 µg/mL gentamicin, and the diluted blood was gently overlaid on sterile Histopaque®-1083 (Sigma-Aldrich) at the ratio of 2∶1 and centrifuged at 800× *g* for 15 min at room temperature. From the multilayer separation, the buffycoat was collected by aspiration and washed thrice with sterile PBS containing 50 µg/mL gentamicin, and cells collected by centrifugation at 250× *g* for 10 min at room temperature. The resulting cell pellets were resuspended in DMEM with 20% FBS and 50 µg/mL gentamicin at concentration of 2×10^6^ viable cells/mL, and then 6 mL aliquots were cultured overnight at 37°C with 5% CO_2_ in 25 cm^2^ poly-D-lysine coated flask, followed by removal of non-adherent cells by washing with sterile PBS. The cell layers were further cultured for 3 days in fresh DMEM, FBS, and gentamicin media as above. Subsequently, gentamicin was removed and the cells were kept in RPMI-1640 media with 20% FBS with no antibiotic for another 2 days prior to use.

### Tissue culture cells and growth conditions

Mouse macrophage cell line RAW264.7 was provided by Dr. Guolong Zhang, Department of Animal Science, Oklahoma State University (ATCC, Manassas, VA; [60]), and dog macrophage cell line DH82 [61] was provided by Dr. Susan E. Little, Department of Veterinary Pathobiology, Oklahoma State University. Both cell lines were cultured at 37°C with 5% CO_2_ tension in RPMI-1640 media with 10% FBS.

### Infection of primary macrophage cells

For infection of primary macrophage cells, *Y. pestis* strain KIM6-2053.1+ was grown in BHI broth at 26°C as described above, and the inocula quantified by OD_600 nm_ as compared with a standard curve for CFU/OD_600 nm_. At the time of infection, aliquots of 6 mL of inocula containing 4.0–5.4×10^6^ *Y. pestis* in RPMI-1640 with 10% FBS media were added to each 25 cm^2^ flask containing 0.8–1.1×10^6^ viable macrophages as trypan blue exclusion assay carried out on the corresponding replica samples, yielding a multiplicity of infection of 5∶1. To initiate the infection at zero time, the flasks were centrifuged at 800× *g* for 3 min to enhance macrophage-bacteria contact and then incubated at 37°C under 5% CO_2_ concentration for 30 min. Subsequently, adherent macrophages were washed gently thrice with 6 to 7 mL of sterile PBS and treated with 6 mL of RPMI-1640 with 10% FBS media containing 50 µg/mL gentamicin for 2 h under the same incubation conditions to kill extracellular bacteria. At the end of gentamicin exposure, macrophages were once again washed gently thrice with 6 to 7 mL of PBS and then 6 mL of antibiotic-free RPMI-1640 with 10% FBS media were added. From the resulting infection, samples were collected for light and transmission electron microscopic studies at various post-infection intervals. At 2.5 h p.i., samples were collected following removal of the gentamicin media and PBS washes by treating with 0.05% trypsin-EDTA (Mediatech Inc., Manassas, VA) at 37°C for 1 to 3 min; however, at 7.5 and 27.5 h p.i., 2 h prior to sampling, the infected adherent macrophages were treated with RPMI-1640 with 10% FBS media and 50 µg/mL of gentamicin for 2 h to kill any extracellular *Y. pestis*.

### Infection of tissue culture cells

To prepare tissue culture cells for infection, RAW264.7 and DH82 cell monolayers grown in 75 cm^2^ tissue culture flasks were released into the fresh RPMI-1640 with 10% FBS media using cell scraper (BD Falcon, Biosciences Discovery Labware, Bedford, MA) and number of cells per unit volume were counted using hemocytometer. Prior to use in assays, tissue culture cell viability was assessed by trypan blue exclusion assay with >95% viability required for subsequent use. Infection of RAW264.7 and DH82 macrophage cell lines with *Y. pestis* KIM6+ or *Y. enterocolitica* were carried out in 96-well flat-bottom plates (FALCON flat bottom polystyrene plate, Becton Dickinson Company). Prior to infection, 100 µL of cell suspensions containing 1×10^6^ RAW264.7 viable cells/mL of RPMI-1640 with 10% FBS were sub-cultured in wells A1 to A3 of the top row of 96-well flat-bottom tissue culture plates and in the same way DH82 cells were sub-cultured in wells A7 to A9. These plates were incubated at 37°C with 5% CO_2_ concentration for 12 to 16 h. For infection, 100 µL of RPMI-1640 with 10% FBS media containing 5×10^6^ *Y. pestis* KIM6+ or *Y. enterocolitica* cultured overnight at 26°C was added to each well of A1–3 and A7–9 of all plates yielding a MOI of 5∶1. The resulting infection was carried out at 37°C with 5% CO_2_ for 30 min and then treated with 50 µg/mL gentamicin for *Y. pestis* or 100 µg/mL gentamicin for *Y. enterocolitica* for 2 h as above mentioned for primary macrophage infections. At 0 h p.i., replica of inocula and tissue culture cells with which the infection had started were taken as such for analysis. For 0.5 and 2.5 h p.i., samples were obtained immediately before adding gentamicin and antibiotic-free media, respectively. At 1.25 and 2.0 h p.i., samples were obtained after removal of the gentamicin media and washing thrice with PBS. Prior to 5.0, 7.5, 12.5, 18.5 and 27.5 h p.i. sampling, the tissue layers were treated for a second time with RPMI-1640 with 10% FBS media contained 50 µg/mL of gentamicin for *Y. pestis* or 100 µg/mL gentamicin for *Y. enterocolitica* for 2 h as mentioned above.

### Infection of tissue culture cells in antibiotic-free media

To determine whether *Y. pestis* intracellular filamentous morphologic change occurred in the absence of gentamicin, RAW264.7 and DH82 cells were infected for 30 min as described above, but instead of treating with gentamicin to kill extracellular bacteria, the adherent macrophages were washed three times with 100 µL of PBS to remove extracellular bacteria and 100 µL of HBSS containing 10% FBS was added to wells. Infected macrophages were then incubated for 2 or 4.5 h and the morphology of intracellular *Y. pestis* observed by light microscopy.

### Determination of colony forming units

CFUs were determined using a novel high-throughput assay developed by Nizet and colleagues and modified for use with *Y. pestis* in which infected tissue culture cells in 96-well plates are lyzed, serial dilution, and microcolonies of released intracellular bacteria are grown in soft agar in the 96-well plates and counted to determine the CFUs [59], [62]. At the time of sampling, media from wells was aspirated, adherent tissue culture cells washed gently thrice with 100 µL of PBS, and the final wash transferred laterally to wells in the A row of the plate. Following washing and transfer of the final wash, 100 µL of 0.1% Triton X-100 in sterile PBS was added to wells A1–3 and A7–9, and the plates were incubated for 10 min at 37°C with 160 rpm on an oscillating shaker to allow lysis and release of intracellular *Y. pestis* into the media. Eighty µL of PBS were added to wells B1 to H12, and the cell lysates and wash solutions in row A were serially diluted 5-fold in PBS by transferring 20 µL from each row to the next from row A to row H. Subsequently, 80 µL of bacterial suspension left in each well of 96-well plate was overlaid gently with 120 µL of 0.83% Bacto-agar (Becton Dickinson, Franklin Lakes, NJ) maintained at 45 to 48°C prior to dispensing. Finally, the plates were incubated at 26°C for 16 to 24 h, and the resulting growth of micro-colonies counted using an inverted light microscope. Before adopting this micro-plate based colony counting, accuracy of the method was compared with conventional CFU determinations using a 10 cm agar plate method, which is described in detail by Wendte *et. al.*, 2011 [59].

### Determination of genomic equivalents

To determine the total number of *Y. pestis* comprised of culturable, viable but non-culturable, and dead bacteria in samples at various post-infection time points, GEs were determined using PCR. A 90 bp fragment from the single copy *Y. pestis* specific ‘*fur*’ gene was amplified using forward primer 5′-TCT GGA AGT GTT GCA AAA TCC TG-3′ and reverse primer, 5′- AAG CCA ATC TCT TCA CCA ATA TCG-3′. PCR reactions were carried out with 300 nM of each primer in 20 µL volume using the Fast SYBR green master mix (Applied Biosystems, Carlsbad, CA) according to the manufacture's instruction (initial enzyme activation at 95°C for 20 sec, followed by 40 cycles of denaturation at 95°C for 3 sec and annealing/extension at 60°C for 30 sec). Standard curves of threshold cycle (ct) values versus bacterial number were prepared by spiking known CFUs of *Y. pestis* strain KIM6+ into samples containing 1×10^6^ RAW264.7 or DH82 cells/mL. The assay was initiated immediately after adding the bacteria into the cell suspension by first heat bursting at 95°C for 10 min, and after centrifugation at 1,000× *g* for 1 min, 2 µL of supernatant was used to determine the ct-value using a 7500 Fast Real-Time PCR system (Applied Biosystems, Carlsbad, CA). For samples containing infected macrophages, the assay was initiated by heat burst, and the resulting samples were assayed as described above.

### Determination of infected macrophage counts

To determine the number of macrophages at a particular infection interval, counts of macrophages were conducted. At each sampling period, infected RAW264.7 and DH82 cells in wells of replica plates were released by gentle pipetting and dilution in 0.4% trypan blue-PBS dye. Using a hemocytometer and microscope, the number of viable and non-viable macrophages was determined.

### Determination of the percent infectivity

At each infection interval, samples of *Y. pestis* infected RAW264.7 and DH82 cells were cytospun (Statspin Cytofuge, Norwood, MA) onto glass microscope slides at low speed for 5 min and stained with Wright Giemsa stain. Using a light microscope at a 1,000× magnification, approximately 400 macrophages per sample were examined to count the number of macrophages which had at least one detectable intracellular bacterium, and from these values, the percent infectivity was calculated.

### Calculation of CFUs and GEs per macrophage

At each infection interval, number of CFUs and GEs per macrophage was calculated from the CFUs, GEs and macrophage number per mL and percent infectivity of the corresponding interval using the formulas:


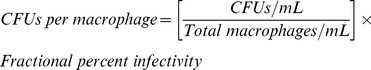



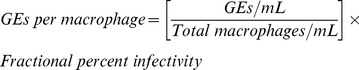


Here, total macrophages/mL represents the sum of trypan blue positive and negative cells per mL at each infection interval.

### Macrophage cytotoxicity assay

Replica samples of RAW264.7 and DH82 cells infected with *Y. pestis* were used for the cytotoxicity assay. At each infection interval, the activity of the eukaryotic cytoplasmic enzyme lactate dehydrogenase (LDH) in the extracellular media was measured as an indicator of macrophage cell lysis. The LDH activity was measured using the CytoTox-ONE™ Homogenous Membrane Integrity Assay kit (Promega, Madison, WI) in a POLAR star OPTIMA (BMG Labtechnologies Inc., Cary, NC) at excitation and emission of 560 and 590 nm, respectively. From these values, percent cytotoxicity was calculated using the formula:


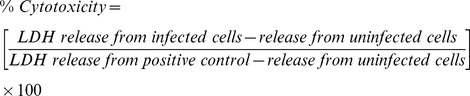


Positive control release is the maximum LDH release from uninfected macrophages induced by cell lysis with 0.1% Triton X-100.

### Microscopic studies

Morphological features of intracellular *Y. pestis* and of infected macrophages were examined using microscopy.

#### Light microscopy

*Y. pestis* infected macrophage cell lines, RAW264.7 and DH82, and primary macrophage isolates from mouse spleen and dog peripheral blood were spun onto microscopic slides at slow speed for 5 min using a cytocentrifuge (Statspin Cytofuge), and the slides stained with Wright Giemsa stain. The stained slides were examined at 1,000× magnification using light microscopy.

#### Transmission electron microscopy

Primary macrophage isolates from mouse spleen and dog peripheral blood and tissue culture cell lines RAW264.7 and DH82 were infected as described above and sampled at 2.5, 7.5 and 27.5 h p.i. During sampling, infected primary macrophage isolates were released into RPMI-1640 with 10% FBS media at the intended sampling intervals by using trypsin as explained elsewhere, then collected at 250× *g* for 10 min, and finally fixed with 2.5% (vol/vol) glutaraldehyde in PBS for 1 h at room temperature. After fixing, the cells were changed into PBS as noted above and kept at 4°C until processing. In case of tissue culture macrophages, infected cells in 25 cm^2^ flasks were fixed therein for 15 min on ice with 2.5% (vol/vol) glutaraldehyde in PBS. Subsequently, the fixed cells were transferred using a CytoOne® cell scraper (USA Scientific Inc., Ocala, FL) into the above noted glutaraldehyde solution to fix further for 45 min at room temperature. Thereafter, the fixed cells were collected by centrifuging at 250× *g* for 10 min at room temperature, suspended in PBS and maintained at 4°C for further processing. For this processing, cells were postfixed with 1% osmium tetroxide (Polysciences Inc., Warrington, PA) in PBS for 1 h. Subsequently, cells washed thrice with PBS were en bloc stained with 1% aqueous uranyl acetate (Ted Pella Inc., Redding, CA) for 1 h, dehydrated in ascending grades of ethanol and then embedded in Eponate resin (Ted Pella Inc.). Ultra-thin sections of 90 nm were prepared using a Leica Ultracut UCT ultramicrotome (Leica Microsystems Inc., Bannock-burn, IL) and stained with uranyl acetate and lead citrate (Sigma-Aldrich). Finally these sections were examined in a JEOL 1,200 EX transmission electron microscopy (JEOL USA Inc., Peabody, MA).

### Morphometric analysis of TEM images

Electron microscopic images were assessed using gimp® photo-editing software version 2.6 (http://www.gimp.org/). Analysis was conducted on 9 or more randomly selected single cell images for each infected cell type and infection interval. Assessment of filamentous bacterial morphology was based on quantitative comparison of the 1 µm measure bar on images to determine the bacterial length. Bacteria longer than 5 µm, double the maximum length of normal *Y. pestis* (0.5–0.8×1–2.5 µm), were considered to be filamentous *Y. pestis*. Bacteria exhibiting normal morphology without intracellular vacuolation or irregular electron dense granulation were scored as intact *Y. pestis*. Intact and filamentous *Y. pestis* per infected RAW264.7 or DH82 cell were quantified based on these criteria. For the infected macrophages, the percentage of cells which had spacious extension of YCV was quantified.

### Statistical analysis

To determine statistical significance for comparison of two means, Student's t-test was used. For comparisons of more than two means, one-way ANOVA and subsequent ‘Tukey HSD’ post hoc methods were employed. The resulting analysis was reported as significant difference at p-value 0.01 or 0.05 for a given comparison.

## References

[1] Perry RD, Fetherston JD (1997) *Yersinia pestis*–etiologic agent of plague. Clin Microbiol Rev 10: 35–66. 8993858 10.1128/cmr.10.1.35 PMC172914

[2] Anonymous (1999) Plague manual–epidemiology, distribution, surveillance and control. Wkly Epidemiol Rec 74: 447–481. 10635759

[3] Cully JF Jr, Johnson TL, Collinge SK, Ray C (2010) Disease limits populations: plague and black-tailed prairie dogs. Vector Borne Zoonotic Dis 10: 7–15. 20158327 10.1089/vbz.2009.0045 PMC2945311

[4] Kartman L, Prince FM, Quan SF, Stark HE (1958) New knowledge on the ecology of sylvatic plague. Ann N Y Acad Sci 70: 668–711. 13559927 10.1111/j.1749-6632.1958.tb35421.x

[5] Orloski KA, Eidson M (1995) *Yersinia pestis* infection in three dogs. J Am Vet Med Assoc 207: 316–318. 7628931

[6] Salkeld DJ, Stapp P (2006) Seroprevalence rates and transmission of plague (*Yersinia pestis*) in mammalian carnivores. Vector Borne Zoonotic Dis 6: 231–239. 16989561 10.1089/vbz.2006.6.231

[7] Gould LH, Pape J, Ettestad P, Griffith KS, Mead PS (2008) Dog-associated risk factors for human plague. Zoonoses Public Health 55: 448–454. 18489541 10.1111/j.1863-2378.2008.01132.x

[8] Boone A, Kraft JP, Stapp P (2009) Scavenging by mammalian carnivores on prairie dog colonies: implications for the spread of plague. Vector Borne Zoonotic Dis 9: 185–190. 18945188 10.1089/vbz.2008.0034

[9] Sebbane F, Gardner D, Long D, Gowen BB, Hinnebusch BJ (2005) Kinetics of disease progression and host response in a rat model of bubonic plague. Am J Pathol 166: 1427–1439. 15855643 10.1016/S0002-9440(10)62360-7 PMC1606397

[10] Sebbane F, Jarrett CO, Gardner D, Long D, Hinnebusch BJ (2006) Role of the *Yersinia pestis* plasminogen activator in the incidence of distinct septicemic and bubonic forms of flea-borne plague. Proc Natl Acad Sci U S A 103: 5526–5530. 16567636 10.1073/pnas.0509544103 PMC1414629

[11] Sha J, Endsley JJ, Kirtley ML, Foltz SM, Huante MB, et al (2011) Characterization of an F1 deletion mutant of *Yersinia pestis* CO92, pathogenic role of F1 antigen in bubonic and pneumonic plague, and evaluation of sensitivity and specificity of F1 antigen capture-based dipsticks. J Clin Microbiol 49: 1708–1715. 21367990 10.1128/JCM.00064-11 PMC3122665

[12] Laws TR, Davey MS, Titball RW, Lukaszewski R (2010) Neutrophils are important in early control of lung infection by *Yersinia pestis* . Microbes Infect 12: 331–335. 20114086 10.1016/j.micinf.2010.01.007

[13] Lukaszewski RA, Kenny DJ, Taylor R, Rees DG, Hartley MG, et al (2005) Pathogenesis of *Yersinia pestis* infection in BALB/c mice: effects on host macrophages and neutrophils. Infect Immun 73: 7142–7150. 16239508 10.1128/IAI.73.11.7142-7150.2005 PMC1273833

[14] Du Y, Rosqvist R, Forsberg A (2002) Role of fraction 1 antigen of *Yersinia pestis* in inhibition of phagocytosis. Infect Immun 70: 1453–1460. 11854232 10.1128/IAI.70.3.1453-1460.2002 PMC127752

[15] Smiley ST (2008) Immune defense against pneumonic plague. Immunol Rev 225: 256–271. 18837787 10.1111/j.1600-065X.2008.00674.x PMC2804960

[16] Cavanaugh DC, Randall R (1959) The role of multiplication of *Pasteurella pestis* in mononuclear phagocytes in the pathogenesis of flea-borne plague. J Immunol 83: 348–363. 13808585

[17] Grabenstein JP, Fukuto HS, Palmer LE, Bliska JB (2006) Characterization of phagosome trafficking and identification of PhoP-regulated genes important for survival of *Yersinia pestis* in macrophages. Infect Immun 74: 3727–3741. 16790745 10.1128/IAI.00255-06 PMC1489716

[18] Pujol C, Grabenstein JP, Perry RD, Bliska JB (2005) Replication of *Yersinia pestis* in interferon gamma-activated macrophages requires *ripA*, a gene encoded in the pigmentation locus. Proc Natl Acad Sci U S A 102: 12909–12914. 16120681 10.1073/pnas.0502849102 PMC1200267

[19] Pujol C, Bliska JB (2003) The ability to replicate in macrophages is conserved between *Yersinia pestis* and *Yersinia pseudotuberculosis* . Infect Immun 71: 5892–5899. 14500510 10.1128/IAI.71.10.5892-5899.2003 PMC201058

[20] Pujol C, Bliska JB (2005) Turning *Yersinia* pathogenesis outside in: subversion of macrophage function by intracellular Yersiniae. Clin Immunol 114: 216–226. 15721832 10.1016/j.clim.2004.07.013

[21] Straley SC, Harmon PA (1984) *Yersinia pestis* grows within phagolysosomes in mouse peritoneal macrophages. Infect Immun 45: 655–659. 6469352 10.1128/iai.45.3.655-659.1984 PMC263345

[22] Straley SC, Harmon PA (1984) Growth in mouse peritoneal macrophages of *Yersinia pestis* lacking established virulence determinants. Infect Immun 45: 649–654. 6469351 10.1128/iai.45.3.649-654.1984 PMC263344

[23] Perry RD, Straley SC, Fetherston JD, Rose DJ, Gregor J, et al (1998) DNA sequencing and analysis of the low-Ca^2+^-response plasmid pCD1 of *Yersinia pestis* KIM5. Infect Immun 66: 4611–4623. 9746557 10.1128/iai.66.10.4611-4623.1998 PMC108568

[24] Lindler LE, Tall BD (1993) *Yersinia pestis* pH 6 antigen forms fimbriae and is induced by intracellular association with macrophages. Mol Microbiol 8: 311–324. 8100346 10.1111/j.1365-2958.1993.tb01575.x

[25] Galyov EE, Smirnov O, Karlishev AV, Volkovoy KI, Denesyuk AI, et al (1990) Nucleotide sequence of the *Yersinia pestis* gene encoding F1 antigen and the primary structure of the protein. Putative T and B cell epitopes. FEBS Lett 277: 230–232. 1702734 10.1016/0014-5793(90)80852-a

[26] Liu F, Chen H, Galvan EM, Lasaro MA, Schifferli DM (2006) Effects of Psa and F1 on the adhesive and invasive interactions of *Yersinia pestis* with human respiratory tract epithelial cells. Infect Immun 74: 5636–5644. 16988239 10.1128/IAI.00612-06 PMC1594889

[27] Ye Z, Kerschen EJ, Cohen DA, Kaplan AM, van Rooijen N, et al (2009) Gr1^+^ cells control growth of YopM-negative *Yersinia pestis* during systemic plague. Infect Immun 77: 3791–3806. 19581396 10.1128/IAI.00284-09 PMC2738001

[28] Gasper PW, Barnes AM, Quan TJ, Benziger JP, Carter LG, et al (1993) Plague (*Yersinia pestis*) in cats: description of experimentally induced disease. J Med Entomol 30: 20–26. 8433327 10.1093/jmedent/30.1.20

[29] Rocke TE, Mencher J, Smith SR, Friedlander AM, Andrews GP, et al (2004) Recombinant F1-V fusion protein protects black-footed ferrets (*Mustela nigripes*) against virulent *Yersinia pestis* infection. J Zoo Wildl Med 35: 142–146. 15305507 10.1638/03-021

[30] Eidson M, Thilsted JP, Rollag OJ (1991) Clinical, clinicopathologic, and pathologic features of plague in cats: 119 cases (1977–1988). J Am Vet Med Assoc 199: 1191–1197. 1752774

[31] Rollag OJ, Skeels MR, Nims LJ, Thilsted JP, Mann JM (1981) Feline plague in New Mexico: report of five cases. J Am Vet Med Assoc 179: 1381–1383. 7341569

[32] Watson RP, Blanchard TW, Mense MG, Gasper PW (2001) Histopathology of experimental plague in cats. Vet Pathol 38: 165–172. 11280372 10.1354/vp.38-2-165

[33] Rust JH Jr, Cavanaugh DC, O'Shita R, Marshall JD Jr (1971) The role of domestic animals in the epidemiology of plague. I. Experimental infection of dogs and cats. J Infect Dis 124: 522–526. 5115673 10.1093/infdis/124.5.522

[34] Underhill DM, Ozinsky A (2002) Phagocytosis of microbes: complexity in action. Annu Rev Immunol 20: 825–852. 11861619 10.1146/annurev.immunol.20.103001.114744

[35] Flannagan RS, Jaumouille V, Grinstein S (2011) The cell biology of phagocytosis. Annu Rev Pathol 7: 49–86. 10.1146/annurev-pathol-011811-132445 21910624

[36] Ponnusamy D, Hartson SD, Clinkenbeard KD (2011) Intracellular *Yersinia pestis* expresses general stress response and tellurite resistance proteins in mouse macrophages. Vet Microbiol 150: 146–151. 21295415 10.1016/j.vetmic.2010.12.025

[37] Perry RD, Mier I Jr, Fetherston JD (2007) Roles of the Yfe and Feo transporters of *Yersinia pestis* in iron uptake and intracellular growth. Biometals 20: 699–703. 17206386 10.1007/s10534-006-9051-x

[38] Bliska JB, Pujol C, Klein KA, Romanov GA, Palmer LE, et al (2009) *Yersinia pestis* can reside in autophagosomes and avoid xenophagy in murine macrophages by preventing vacuole acidification. Infect Immun 77: 2251–2261. 19289509 10.1128/IAI.00068-09 PMC2687347

[39] Lorian V, Atkinson B (1975) Abnormal forms of bacteria produced by antibiotics. Am J Clin Pathol 64: 678–688. 242211 10.1093/ajcp/64.5.678

[40] Spinner JL, Seo KS, O'Loughlin JL, Cundiff JA, Minnich SA, et al (2010) Neutrophils are resistant to *Yersinia* YopJ/P-induced apoptosis and are protected from ROS-mediated cell death by the type III secretion system. PLoS One 5: e9279. 20174624 10.1371/journal.pone.0009279 PMC2823771

[41] Thomson NR, Howard S, Wren BW, Prentice MB (2007) Comparative genome analyses of the pathogenic Yersiniae based on the genome sequence of *Yersinia enterocolitica* strain 8081. Adv Exp Med Biol 603: 2–16. 17966400 10.1007/978-0-387-72124-8_1

[42] Rust JH Jr, Miller BE, Bahmanyar M, Marshall JD Jr, Purnaveja S, et al (1971) The role of domestic animals in the epidemiology of plague. II. Antibody to *Yersinia pestis* in sera of dogs and cats. J Infect Dis 124: 527–531. 5115674 10.1093/infdis/124.5.527

[43] Vernati G, Edwards WH, Rocke TE, Little SF, Andrews GP (2011) Antigenic profiling of *Yersinia pestis* infection in the Wyoming coyote (*Canis latrans*). J Wildl Dis 47: 21–29. 21269993 10.7589/0090-3558-47.1.21

[44] Justice SS, Hunstad DA, Seed PC, Hultgren SJ (2006) Filamentation by *Escherichia coli* subverts innate defenses during urinary tract infection. Proc Natl Acad Sci U S A 103: 19884–19889. 17172451 10.1073/pnas.0606329104 PMC1750882

[45] Ogawa M, Takade A, Miyamoto H, Taniguchi H, Yoshida S (2001) Morphological variety of intracellular microcolonies of *Legionella* species in Vero cells. Microbiol Immunol 45: 557–562. 11529563 10.1111/j.1348-0421.2001.tb02658.x

[46] Chauhan A, Madiraju MV, Fol M, Lofton H, Maloney E, et al (2006) *Mycobacterium tuberculosis* cells growing in macrophages are filamentous and deficient in FtsZ rings. J Bacteriol 188: 1856–1865. 16484196 10.1128/JB.188.5.1856-1865.2006 PMC1426569

[47] Rosenberger CM, Gallo RL, Finlay BB (2004) Interplay between antibacterial effectors: A macrophage antimicrobial peptide impairs intracellular *Salmonella* replication. Proc Natl Acad Sci U S A 101: 2422–2427. 14983025 10.1073/pnas.0304455101 PMC356966

[48] Rosenberger CM, Finlay BB (2002) Macrophages inhibit *Salmonella typhimurium* replication through MEK/ERK kinase and phagocyte NADPH oxidase activities. J Biol Chem 277: 18753–18762. 11821396 10.1074/jbc.M110649200

[49] Henry T, Garcia-del Portillo F, Gorvel JP (2005) Identification of *Salmonella* functions critical for bacterial cell division within eukaryotic cells. Mol Microbiol 56: 252–267. 15773994 10.1111/j.1365-2958.2005.04540.x

[50] Schapiro JM, Libby SJ, Fang FC (2003) Inhibition of bacterial DNA replication by zinc mobilization during nitrosative stress. Proc Natl Acad Sci U S A 100: 8496–8501. 12829799 10.1073/pnas.1033133100 PMC166257

[51] Romberg L, Levin PA (2003) Assembly dynamics of the bacterial cell division protein FtsZ: poised at the edge of stability. Annu Rev Microbiol 57: 125–154. 14527275 10.1146/annurev.micro.57.012903.074300 PMC5517307

[52] Bi E, Lutkenhaus J (1993) Cell division inhibitors SulA and MinCD prevent formation of the FtsZ ring. J Bacteriol 175: 1118–1125. 8432706 10.1128/jb.175.4.1118-1125.1993 PMC193028

[53] Imlay JA, Linn S (1987) Mutagenesis and stress responses induced in *Escherichia coli* by hydrogen peroxide. J Bacteriol 169: 2967–2976. 3298208 10.1128/jb.169.7.2967-2976.1987 PMC212335

[54] Justice SS, Hunstad DA, Cegelski L, Hultgren SJ (2008) Morphological plasticity as a bacterial survival strategy. Nat Rev Microbiol 6: 162–168. 18157153 10.1038/nrmicro1820

[55] Fukuto HS, Svetlanov A, Palmer LE, Karzai AW, Bliska JB (2010) Global gene expression profiling of *Yersinia pestis* replicating inside macrophages reveals the roles of a putative stress-induced operon in regulating type III secretion and intracellular cell division. Infect Immun 78: 3700–3715. 20566693 10.1128/IAI.00062-10 PMC2937462

[56] Chen K, Sun GW, Chua KL, Gan YH (2005) Modified virulence of antibiotic-induced *Burkholderia pseudomallei* filaments. Antimicrob Agents Chemother 49: 1002–1009. 15728895 10.1128/AAC.49.3.1002-1009.2005 PMC549247

[57] Bearden SW, Fetherston JD, Perry RD (1997) Genetic organization of the yersiniabactin biosynthetic region and construction of avirulent mutants in *Yersinia pestis* . Infect Immun 65: 1659–1668. 9125544 10.1128/iai.65.5.1659-1668.1997 PMC175193

[58] Conchas RF, Carniel E (1990) A highly efficient electroporation system for transformation of *Yersinia* . Gene 87: 133–137. 2332165 10.1016/0378-1119(90)90505-l

[59] Wendte JM, Ponnusamy D, Reiber D, Blair JL, Clinkenbeard KD (2011) *In vitro* efficacy of antibiotics commonly used to treat human plague against intracellular *Yersinia pestis* . Antimicrob Agents Chemother 55: 3752–3757. 21628541 10.1128/AAC.01481-10 PMC3147644

[60] Raschke WC, Baird S, Ralph P, Nakoinz I (1978) Functional macrophage cell lines transformed by Abelson leukemia virus. Cell 15: 261–267. 212198 10.1016/0092-8674(78)90101-0

[61] Wellman ML, Krakowka S, Jacobs RM, Kociba GJ (1988) A macrophage-monocyte cell line from a dog with malignant histiocytosis. In Vitro Cell Dev Biol 24: 223–229. 3350786 10.1007/BF02623551

[62] Nizet V, Smith AL, Sullam PM, Rubens CE (1998) A simple microtiter plate screening assay for bacterial invasion or adherence. Methods Cell Sci 20: 107–111.

